# A Review of Grain Refinement and Texture Engineering in Aluminum Alloy Magnetron Sputtering Targets

**DOI:** 10.3390/ma18143235

**Published:** 2025-07-09

**Authors:** Run-Xin Song, Dong Wang, Yiqiao Yang, Jinjiang He, Song Li, Hai-Le Yan, Liang Zuo

**Affiliations:** 1 Key Laboratory for Anisotropy and Texture of Materials (Ministry of Education), School of Material Science and Engineering, Northeastern University, Shenyang 110819, China; srunxin@yeah.net (R.-X.S.); lis@atm.neu.edu.cn (S.L.); lzuo@mail.neu.edu.cn (L.Z.); 2 College of Mechanical Engineering, Zhejiang College of Zhejiang University of Technology, Shaoxing 312030, China; 3 Analytical and Testing Center, Northeastern University, Shenyang 110819, China; yangyq@mail.neu.edu.cn; 4 GRIKIN Advanced Materials Co., Ltd., Beijing 102200, China; hejinjiang@grikin.com; 5 National Engineering Research Center for Key Materials of Integrated Circuits, Beijing 100088, China

**Keywords:** aluminum alloy target, magnetron sputtering, grain refinement, texture engineering

## Abstract

Aluminum and its alloy magnetron sputtering targets, owing to their superior electrical/thermal conductivity and robust substrate adhesion, serve as critical materials in advanced electronics and information technologies. It is known that the microstructure of the target, including grain uniformity and crystallographic texture, directly affects the sputtering performance and the quality of the deposited thin film. Despite extensive research efforts, the review paper focused on the microstructure of aluminum target materials is still absent. In that context, the recent progress on the Al alloy target is reviewed, focusing on grain refinement and texture control strategies. The roles of alloying elements, such as Si, Cu, and rare-earth Sc and Nd, are described first. The two conventional manufacturing techniques of fabricating Al targets, including melting and powder metallurgy, are introduced. Then, studies on grain refinement by thermomechanical processing routes (hot/cold rolling, annealing and forging) are summarized. Lastly, texture engineering through deformation and heat treatment protocols (unidirectional/multidirectional rolling, deformation thickness, and composite deformation modes) is reviewed. By establishing the relationship between thermomechanical processing and microstructure, this review provides insights for designing high-performance aluminum targets tailored to next-generation advanced thin-film applications.

## 1. Introduction

**Figure 1:**
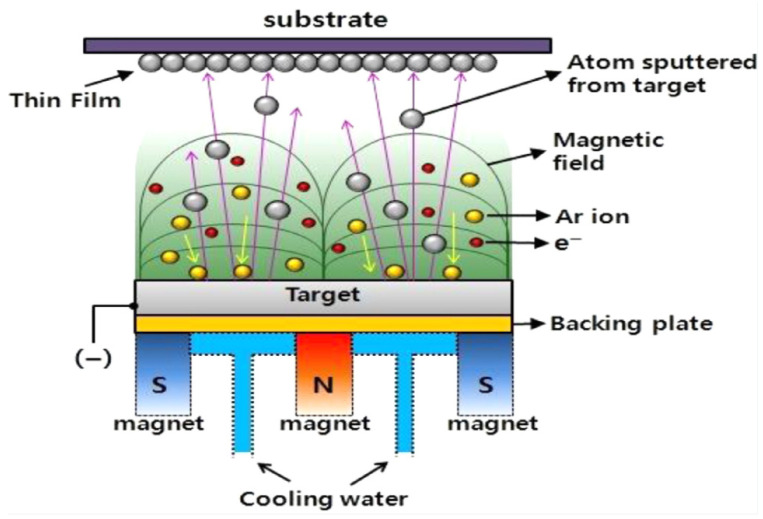
Schematic diagram for the principle of magnetron sputtering [1]. Reprinted from Janarthanan and Thirunavukkarasu (2021), with permission from Elsevier.

Magnetron sputtering is one of the main techniques for preparing thin-film materials, widely used in the electronics and information industries, including integrated circuits, information storage, and liquid crystal displays. The magnetron sputtering technique utilizes ions generated by an ion source, which are accelerated and concentrated in a vacuum to form a high-speed ion beam. This ion beam bombards a solid surface, causing energy transfer between the ions and the atoms on the solid surface, which leads to the ejection of atoms from the solid and their deposition onto a substrate (Figure 1) [1]. The solid material being bombarded, known as the magnetron sputtering target [2,3], serves as the target material for thin-film deposition via the magnetron sputtering method. Aluminum and its alloy target materials, due to their advantages such as excellent thermal/electrical conductivity, favorable processing properties, good adhesion and bonding properties with substrates, and low cost, find extensive applications in the field of electronics and information industries such as wear-resistant materials and high-temperature corrosion resistance [4,5]. The development and use of aluminum alloy targets have become one of the key trends in the sputtering target market.

Aluminum sputtering targets can be categorically classified into three primary types: high-purity aluminum, aluminum alloy, and aluminum compound targets [6]. Extensive investigations have shown that the material factors influencing the quality of sputtering targets include purity, grain morphology, grain size and distribution, and crystallographic texture [7]. Particularly, microstructural characteristics such as grain size and crystallographic texture have been identified as critical determinants of magnetron sputtering efficiency and resultant film quality. Experimental evidence demonstrates that maintaining grain dimensions within an optimized range enables the achievement of both enhanced deposition rates and superior thickness uniformity in deposited films. Furthermore, crystallographic texture has been shown to significantly influence sputtering yield and subsequent microstructure of sputtered film through its modulation of preferential sputtering directions [8]. While these microstructural parameters have been recognized as crucial performance indicators of sputtering targets, the current literature exhibits a conspicuous imbalance in review coverage. Existing attention has predominantly focused on high-purity aluminum targets, as evidenced by recent reviews addressing their fabrication methodologies and industrial applications [9]. Nevertheless, notable gaps persist in a systematic review of aluminum alloy/compound targets and the microstructural engineering of aluminum-based targets. Specifically, there remains a critical absence of comprehensive reviews addressing the control mechanisms for grain size refinement and texture optimization. This knowledge gap underscores the necessity for a thorough examination of current advancements and challenges in this specialized field.

To fill this gap, the recent progress on aluminum sputtering targets with a focus on grain refinement and texture control is critically reviewed. First, the categories of aluminum targets, including high-purity Al, Al-Si, Al-Cu, and rare earth (RE) containing Al-RE system, are introduced (Section 2). The roles of different alloying elements on the Al target are highlighted. Second, the conventional preparation and processing techniques for aluminum targets, focusing on melting–casting and powder metallurgy techniques, are introduced (Section 3). Third, the effects of thermomechanical processing, including forging, hot rolling, cold rolling, and heat treatment, on grain size refinement are reviewed. Last, the typical crystallographic textures in aluminum alloy targets and the advances in texture engineering through thermomechanical processing (unidirectional/multidirectional rolling, deformation thickness, and composite deformation modes) are discussed (Section 4). By integrating this information, this work aims to provide process–microstructure relationships to guide the development of advanced aluminum alloy targets.

## 2. Aluminum and Its Alloy Sputtering Targets

### 2.1. High-Purity Aluminum Target

**Figure 2:**
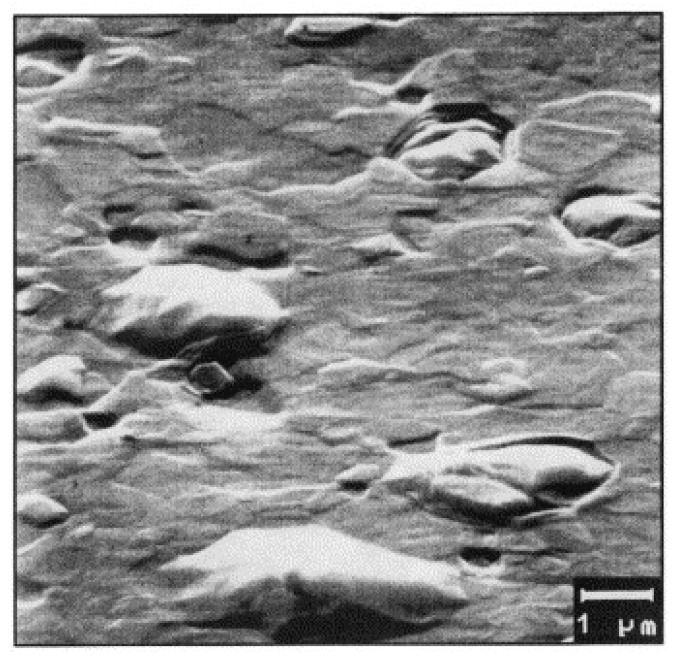
Hillock on magnetron sputtered Al film examined by SEM [12]. Reprinted from Zaborowski and Dumania (2000), with permission from Elsevier.

High-purity aluminum is the most widely used sputtering target material. With the development of large-scale integrated circuits, a series of issues have emerged in the application of high-purity aluminum targets [10]. The most detrimental phenomenon affecting film quality stems from stress-induced migration. Stress migration occurs due to the large differences in thermal expansion coefficients between different components of the integrated circuit, leading to varying levels of stress in each component after the sputtering process. These stress differentials promote the thermal diffusion of excess vacancies, which, through diffusion and aggregation, generate defects such as voids, whiskers, small hills (Figure 2), and structural delamination, severely affecting the quality of the sputtered film [11].

### 2.2. Al-Si Sputtering Target

**Figure 3:**
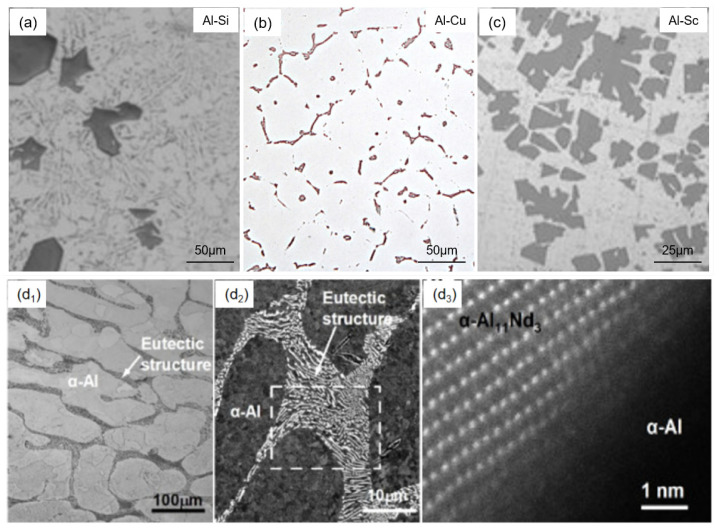
(**a**) Microstructure of cast Al-Si alloy [16]. Reprinted from Manani and Pradhan (2022), with permission from Elsevier. (**b**) Structures of an Al-Cu alloy [17]. Reprinted from Schöbel and Fernández (2019), with permission from Elsevier. (**c**) Optical image of Al-10Sc alloy [18]. Reprinted from J.J. He and Q. Jian (2024), with permission from MDPI. (**d_1_**–**d_3_**) Optical image, backscattered electron image, and high-angle circular dark-field scanning transmission electron microscope (HAADF-STEM) images of the Al-3wt%Nd target alloy [19]. Reprinted from X.Q. Wang and R.X. Song (2025), with permission from Elsevier.

To address the issues encountered with high-purity aluminum targets, it was found that adding a small amount of silicon to high-purity aluminum significantly improves its resistance to stress migration [13]. Microstructural analysis revealed that silicon predominantly exists as discrete eutectic particles (Al-Si phase, Figure 3a) due to its limited solid solubility (<1.65 at.% at eutectic temperature) in the aluminum matrix. These silicon precipitates act as effective grain boundary pinning agents, improving the resistance to stress migration. Furthermore, the existence of silicon precipitates can also suppress abnormal grain growth and promote microstructure refinement, ultimately enhancing target density and sputtering uniformity [14]. In addition, it is reported that the addition of silicon can also increase the recrystallization temperature and hardness, effectively reducing scratch susceptibility during handling and sputtering operations. By means of molecular dynamics simulation, the thermodynamic and transport properties of the Al-Si system were studied by using an angular-dependent potential [15]. It was found that the Al and Si atoms possess almost the same diffusion capacity with the diffusion activation energies of 0.209 eV and 0.202 eV, laying a theoretical foundation for process optimization and performance regulation of the Al-Si target alloy.

### 2.3. Al-Cu Sputtering Targets

To extend the operational lifetime of high-purity aluminum and Al-Si alloy targets, copper alloying has been strategically employed to leverage its grain boundary stabilization effects [20]. During thermal processing, copper atoms undergo thermally activated segregation at grain boundaries (Figure 3b), forming Guinier–Preston (GP) zones and coherent θ′-Al_2_Cu precipitates. These secondary phases synergistically impede dislocation movement through combined chemical and mechanical interactions, achieving dual microstructural benefits, i.e., the enhancement of grain boundary cohesion and the refinement of substructure homogeneity. This multiphase strengthening mechanism suppresses vacancy aggregation pathways, thereby improving stress migration resistance by optimizing vacancy diffusion kinetics [21]. Moreover, by first-principles calculations, it was found that the Al_2_Cu phase exhibits excellent inherent ductility with Pugh’s ratio (B/G) of 2.8 [22]. Thus, the precipitation of Al_2_Cu would not deteriorate mechanical properties.

### 2.4. Al-RE Sputtering Targets

To meet the requirements for larger display sizes and lower resistivity, various methods [23,24], such as alloying and microstructure refinement, have been employed to address the formation of small hills on the thin-film surface. Among them, the alloying of aluminum with rare-earth (such as Sc and Nd) or transition elements is considered one of the most effective approaches to reducing defect formation [23].

Scandium (Sc), a metallic element exhibiting dual characteristics of rare earth and transition metals, remarkably refines aluminum alloy grain structures through trace additions. At present, Sc stands as one of the most potent alloying elements for enhancing aluminum alloy performance [25]. Al-Sc alloys demonstrate superior strength-to-weight ratios, exceptional corrosion resistance, high-temperature stability, and favorable weldability, enabling their widespread application in aerospace, nuclear engineering, high-speed transportation, and advanced sporting equipment [24]. Particularly in microelectronics and information technology, Al-Sc alloy sputtering targets (Figure 3c) achieve notable improvements in electromigration resistance and stress mitigation while retaining comparable electrical conductivity, presenting substantial research and commercial potential for advanced electronic applications [25]. In the Al-Sc target alloys, there exist three intermetallics: Al_3_Sc, Al_2_Sc, and AlSc. By ab initio molecular dynamics and first-principles calculations, it was found that Al_3_Sc and Al_2_Sc are brittle at both ground state and finite temperatures, while AlSc possesses a significantly superior ductility, and the ductility of all Al_3_Sc, Al_2_Sc, and AlSc improves significantly with the elevated temperature [26], which provides a crucial foundation for the design of advanced Al–Sc target alloys.

Neodymium (Nd) stands as another widely utilized alloying element to improve hill-resistance performance in Al target materials. Due to the low electrical resistivity and inhibited hillock formation during thermal processing, Al-Nd alloy targets demonstrate significant advantages in high-resolution, large-scale liquid crystal display manufacturing [27]. The homogeneously distributed Al_11_Nd_3_ intermetallic phases (Figure 3(d_1_–d_3_)) effectively refine grain structures and enhance sputtered film quality through dispersion-strengthening mechanisms. It is found that the microstructural evolution of Al-Nd alloy films exhibits Nd-content dependency [28]. Within the critical Nd concentration range of 2.0–6.0 at.%, complete suppression of hillock formation occurs, facilitating the development of stable solid solutions with quasi-polycrystalline configurations that ensure superior interfacial stability [28]. By first-principles calculations and chemical bonding analysis, it was found that Al_11_Nd_3_ has two types of chemical bonds: the Nd-Al and Al-Al bonds [19]. The Nd-Al bond is a typical ionic bond with electron transfer from Nd to Al, and the Al-Al bond dominated by both *3s*-*3p* and *3p*-*3p* interactions is a metallic (or weak covalent) bond. The mixed ionic and covalent bonds of Al_11_Nd_3_ make this compound a high hardness-brittleness.

## 3. Processing and Preparation Methods

Fabrication techniques of aluminum magnetron sputtering targets are primarily classified into two distinct methodologies based on manufacturing processes: melting–casting techniques and powder metallurgy approaches. As is known, the preparation parameters significantly affect the microstructure and defect features of the produced target materials, which play critical roles in the sputtered film quality. The microstructure features, including the distribution of grain size and crystallographic orientation, decide the deposition rate and uniformity of the deposited thin films. In addition, the defects of sputtering targets (e.g., impurities, cracks, and porosity) also affect the properties of deposited thin films. Higher target purity (with lower total impurity content) yields superior film quality and performance [2]. The cracks and porosity not only reduce the deposition rate and induce the discharge phenomenon but also influence the electrical and optical properties of sputtered films. Thus, stringent control must be implemented over thermal processing parameters and post-sintering forming operations to guarantee the microstructure and performance of the final sputtering targets.

### 3.1. Melting and Casting

For high-purity aluminum targets, in general, the melting method involves melting and casting in a vacuum. Compared to alloys prepared by powder methods, melting alloy targets have lower impurity content and can achieve higher density and larger sizes. Common melting methods include semi-continuous casting, electromagnetic casting, vacuum induction melting, vacuum arc melting, and vacuum electron beam melting. For two or more metals with significantly different melting points and densities, conventional melting methods are generally difficult to achieve homogeneous alloy targets [29]. Vacuum induction melting is a commonly used method for preparing high-purity aluminum targets [30], high-purity low-Sc Al-Sc alloy targets [31], and high-density Al-Nd alloy targets [32].

### 3.2. Powder Metallurgy

For the conventional melting–casting method, it is inadequate for refractory metal targets, such as high-Sc Al-Sc targets, due to the high melting points of alloying elements, such as Sc. Powder metallurgy successfully overcomes these technical limitations through solid-state processing. The fabrication of magnetron sputtering targets by powder metallurgy predominantly employs three established techniques: hot pressing (HP), vacuum hot pressing (VHP), and hot isostatic pressing (HIP) [33]. The powder metallurgy approach demonstrates distinct advantages in achieving homogeneous fine-grained microstructures, minimizing material waste, and maintaining enhanced production efficiency, thereby constituting a principal manufacturing route for advanced sputtering targets [34]. In Al-Si alloy target preparation, the inherent low melting point of aluminum facilitates effective hot-press consolidation. Silicon particulates undergo thermal diffusion bonding within the aluminum matrix under controlled pressure-temperature conditions, enabling precise compositional control in bulk target fabrication [35].

## 4. Grain Refinement

**Figure 4:**
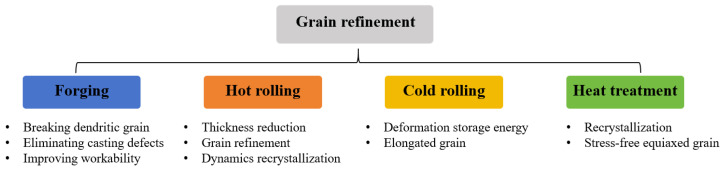
Conventional thermomechanical methods to refine grains for aluminum alloy targets.

Aluminum alloy sputtering targets typically exhibit polycrystalline structures with grain size distributions spanning micrometer to millimeter scales. It is reported that the targets with refined grain dimensions demonstrate enhanced sputtering deposition rates relative to coarse-grained counterparts, while uniform distributions of grain size promote superior film thickness uniformity in deposited film [4]. To achieve aluminum alloy targets with optimized performance characteristics, microstructural refinement is systematically implemented through controlled thermomechanical processing routes [36], such as forging, hot/cold rolling, and heat treatment (Figure 4).

### 4.1. Forging

Forging can effectively eliminate coarse-cast dendrites, mitigate internal material defects, promote grain refinement, and enhance microstructural homogeneity. L. J. Yao and co-workers [37] achieved exceptional microstructural control through three successive forging–elongation and heat treatment cycles, producing Al-Si target materials with uniform grain structures and a constrained grain size below 18 μm, while eliminating abnormal grain growth phenomena. M.X. Liu and co-workers [38] employed multidirectional forging on high-purity Cu-Al alloy ingots, effectively transforming the initial coarse dendritic structure into a refined and homogeneous grain morphology while eliminating internal casting defects. This processing route significantly enhanced the microstructural characteristics and mechanical properties of the alloy ingots, thereby improving their subsequent workability. Following multidirectional forging, the rolling process accompanied by heat treatment is adopted, which further refines the grain structure and enhances its uniformity throughout the ingot.

### 4.2. Hot Rolling

**Figure 5:**
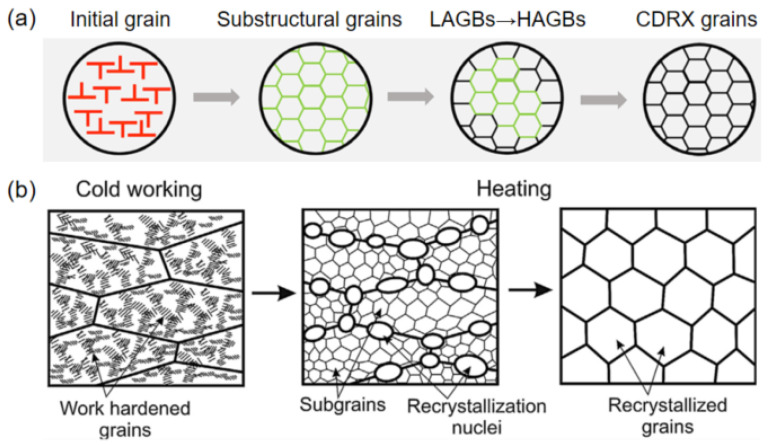
Schematic diagram of grain refinement mechanism of Al alloy target. (**a**) Continuous dynamic recrystallization during hot rolling [39]. Reprinted from Q.J. Wang and X.L. Ren (2022), with permission from Wiley. (**b**) Static recrystallization during cold rolling and annealing [43]. Reprinted from T. Sakai and A. Belyakov (2014), with permission from Elsevier.

Hot rolling constitutes a critical thermomechanical processing technique for achieving grain refinement in aluminum alloy sputtering targets by dynamic recrystallization, as illustrated in Figure 5a. Owing to the high stacking fault energy of Al, the substructural grains with low-angle grain boundary (LAGB) would form during hot rolling due to the effective dynamic recovery. With deformation, the LAGBs transform into high-angle grain boundaries (HAGBs), and continuous dynamic recrystallization (CDRX) grains form, leading to grain refinement [39]. It is demonstrated that the high-purity aluminum subjected to 95% hot rolling deformation at 250 °C followed by 280 °C and 45 min annealing produces refined equiaxed grains with an average grain size of 62.4 μm [40]. The studies on Al-Nd alloys reveal enhanced refinement efficiency, where 87% hot rolling at 350 °C combined with annealing at 250 °C for 4 h achieves an average grain size of 25 μm [41]. The small grain size of the Al-Nd alloys, apart from the thermomechanical processing, could also be attributed to the significant promotion of the addition of Nd in grain refinement. One reason is the solid solution of Nd in aluminum, which reduces the stacking fault energy of aluminum. The other reason could be attributed to the precipitation of Al_11_Nd_3_ which can promote dynamic recrystallization nucleation and pin grain boundaries during deformation. Meanwhile, the eutectic aggregates at grain boundaries exert a drag effect on dislocation motion and grain boundary migration, effectively hindering boundary movement and contributing to grain refinement [42].

### 4.3. Cold Rolling

Cold rolling, as a room-temperature processing technique distinct from hot rolling, enables superior grain refinement in aluminum alloys. Different from the CDRX mechanism during hot rolling, the grain refinement in the Al target realized cold rolling combined with heat treatment is mainly attributed to the discontinuous static recrystallization (DSRX) [43], as illustrated in Figure 5b. During the cold rolling, the elongated work-hardened grains with high deformation storage energy are formed. At the early stage of annealing, static recovery, which is responsible for the formation of recrystallization nuclei as fine dislocation-free crystallites near the grain boundary, occurs. Driven by the stored deformation energy associated with the dislocations and sub-boundaries produced during cold rolling, the recrystallization grains grow by means of the long-range migration of the boundaries, forming the equiaxial fine grains [44]. In addition, in some aluminum alloys with dispersioids, continuous static recrystallization (CSRX) behavior, realized by the gradual growth of subgrain accompanied by particle coarsening, is also observed [45].

**Figure 6:**
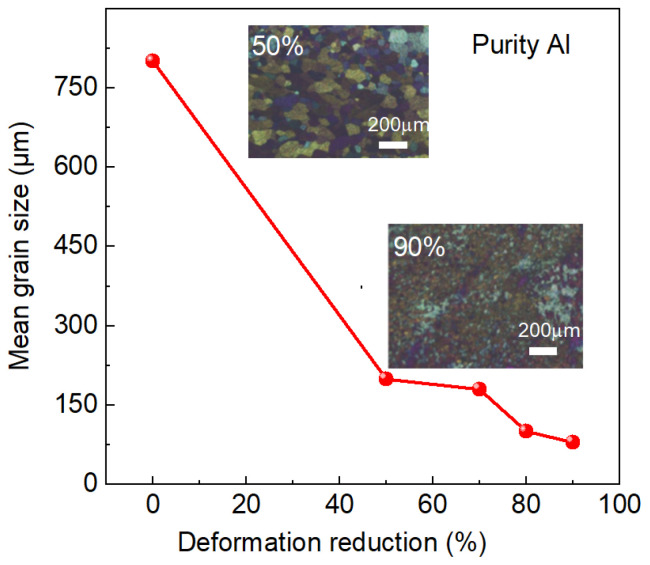
The grain sizes of the cold-rolled high-purity aluminum target with respect to deformation reduction plotted with data extracted from Ref. [48]. The insets are typical microstructures at cold rolled deformation of 50% and 90%.

Experimental studies demonstrate that high-purity aluminum subjected to a cold rolling deformation of 40% followed by annealing at 330 °C for 10 min achieves grain sizes below 200 μm [46]. For the ultra-high-purity Al-0.5wt%Cu specimens, a homogeneous microstructure with average grain sizes of 48 μm is achieved when it is subjected to a cold rolling deformation of 90% and subsequent annealing at 280 °C for 60 min [47]. Furthermore, a deformation-dependent refinement behavior was reported in high-purity aluminum targets [48]. As the deformation reduction is increased, the grain size gradually decreases. At a deformation amount of 50%, the grain size after recrystallization was around 200 μm. At 70% deformation, the grain size was less than 200 μm, and at 80% deformation, the grain size was around 100 μm. Notably, the specimens with a cold roll of 90% attain uniform fine grains (<100 μm) through annealing at 250 °C for 10 min (Figure 6). Moreover, it is found that as the deformation amount increases, the recrystallization temperature decreases. The optimal annealing conditions for recrystallization at deformation amounts of 50%, 70%, 80%, and 90% were found to be 350 for 10 min, 330 °C for 20 min, 300 °C for 10 min, and 250 °C for 10 min [48]. This inverse correlation between deformation magnitude and required annealing temperature indicates that strain energy accumulation acts as the critical factor for driving recrystallization in cold-rolled aluminum systems.

### 4.4. Heat Treatment

Recrystallization annealing critically influences grain size in aluminum alloy targets, with annealing temperature exerting a greater impact than duration time. Experimental investigations on Al-Si systems reveal that materials processed through 520 °C/6 h solution treatment followed by 77% cold rolling and 350 °C/9 h annealing achieve refined microstructures with an average grain size of 15.8 μm [49]. In this study, it is identified that the size and distribution of the second phase in the alloy target directly affect the quality of the sputtered film. Specifically, the segregation of coarse second phases can lead to a decrease in the compositional uniformity of the film, an increase in grain size, and, consequently, a deterioration in the film’s performance. Furthermore, a study on Al-Si alloy subjected to homogenization annealing at 530 °C for 7 h, 70% cold rolling, and recrystallization annealing at 300 °C for 3 h demonstrated a controlled grain growth with an average grain size of 55 μm [50]. Microstructural analysis confirms that annealing above 420 °C significantly increases second-phase solubility, thereby compromising Zener pinning efficacy at grain boundaries and triggering rapid grain coarsening through diminished boundary mobility restriction. These findings establish critical temperature thresholds for maintaining microstructure stability during target processing.

**Figure 7:**
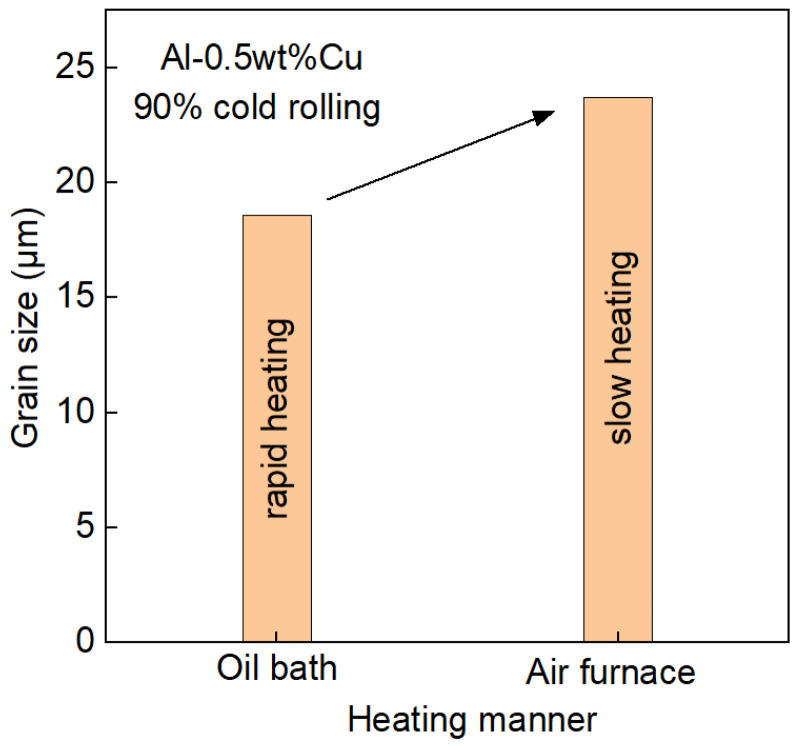
Effect of heating rate on the mean grain sizes for the Al-0.5wt%Cu target with a cold-rolled deformation of 90% plotted with extracted from Ref. [51].

In addition to annealing temperature and duration time, the heating rate was reported as another critical parameter to achieve grain refinement in aluminum alloy targets. H. Zhao and co-workers [51] conducted annealing on ultra-high-purity Al-0.5wt%Cu with a 90% rolling deformation using two different heating methods—slow heating (air furnace) and rapid heating (oil bath)—and identified that the cooling rate significantly alters recrystallization behavior. It is found that the rapid thermal annealing reduces recrystallization initiation temperature by 15–20 °C and accelerates nucleation kinetics, yielding finer recrystallized grains (18.6 ± 13.7 μm) compared to slow-heated counterparts (23.7 ± 17.5 μm), as demonstrated in Figure 7. This heating-rate dependence confirms that accelerated thermal processing enhances both grain refinement efficiency and microstructural homogeneity through controlled recrystallization dynamics.

## 5. Crystallographic Texture Control

**Figure 8:**
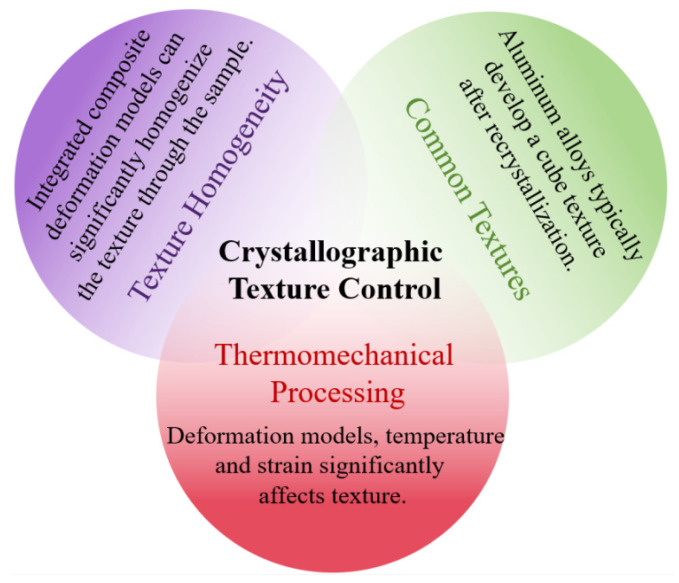
Research progresses on crystallographic textures of Al target.

Due to anisotropic sputtering yields across different atomic planes, crystallographic orientation significantly influences magnetron sputtering performance [52]. The preferential sputtering of specific crystallographic surfaces enables the optimization of deposition rates through targeted crystallographic texture modification. Crucially, the grain orientation distribution within the target governs the angular distribution of sputtered atoms, directly determining thickness uniformity across deposited films. Achieving tailored crystallographic textures requires sophisticated control of thermomechanical processing protocols [53,54,55]. Target fabrication must integrate orientation-specific forming techniques (e.g., texture-controlled rolling) with precisely calibrated annealing cycles to develop and stabilize desired orientation distributions. In this work, the common textures, the effects of routine thermomechanical processing techniques on texture and the methods of homogenizing texture in the Al target are summarized (Figure 8).

### 5.1. Common Textures in Aluminum Alloys

**Figure 9:**
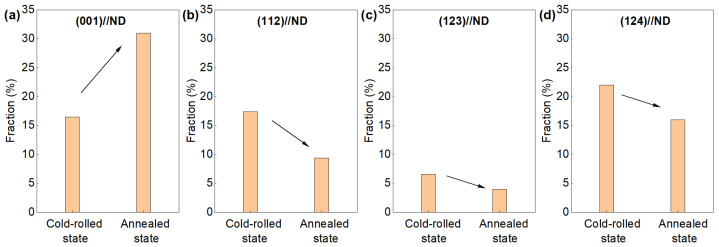
Comparison of texture components at the cold-rolled and annealed states for the high-purity Al target produced with data extracted from Ref. [57]. (**a**) (001)//ND; (**b**) (112)//ND; (**c**) (123)//ND; (**d**) (124)//ND.

Thermomechanical processing induces crystallographic texture in aluminum alloy targets. During rolling, the typical deformation textures of face-centered cubic (fcc) metals, including the Copper texture ({112}<111>), the S texture ({123}<463>), the Brass texture ({011}<211>) and the Goss texture ({011}<100>), were formed, which is ascribed to the dislocation systems of fcc metals and the strain state of rolling [53]. The specific type of deformation texture is closely related to the deformation amount and the initial texture. During the heat treatment following cold rolling, recrystallization occurs with the grains predominantly by the Cube-type ({001}<100>) texture. The formation of the Cube-type recrystallization texture is mainly ascribed to the orientation dependence of grain boundary motion [54]. According to the theory of directional growth, the grain boundary migration rate of 40°<111> is the fastest, which is beneficial to the formation of the Cube-type in the recrystallization process. It is reported that the post-rolling annealing of ultra-high-purity Al (99.999%) processed through the hot rolling of 80% at 50 °C followed by cold rolling of 70% and subsequent recrystallization annealing at 240 °C for 3 h produces fine-grained structures (85 μm) with strong preferential orientation of {100}//ND (ND represents the normal plane) (65% texture fraction) [56]. The investigations by F. Li and co-workers [57] revealed that increasing cold-rolling deformation enhances the dominance of {001} orientation in annealed microstructures while suppressing the texture components of {112}, {123}, and {124} (Figure 9). During annealing, the {001}-oriented grains preferentially nucleate within deformation zones, subsequently consuming adjacent deformed matrix through strain-induced boundary migration. In addition, the orientations of grains in the original dynamic recrystallized structure remained largely unchanged, and the size of dynamic recrystallized grains with other orientations changed little after annealing. In addition, a temperature-dependent texture transition was reported [58]. Under low-temperature rolling, most of the grains in the dynamic recrystallization structure of high-purity aluminum were {001} oriented, showing a clear preferred orientation. As the rolling temperature increased, the Cube-type texture gradually replaced the deformation texture [58], and the texture components of the dynamic recrystallization structure became more diversified. This temperature–texture relationship underscores the critical balance between deformation storage energy and thermal activation in controlling final orientation distributions.

### 5.2. Effect of Thermomechanical Processing on Texture

Apart from conventional unidirectional rolling, multidirectional rolling is also adopted in the industry. Compared with the unidirectional rolling, the strain state of the multidirectional rolling sample is notably distinct, which further affects the development of crystallographic texture. Electron backscatter diffraction (EBSD) analysis showed that the Al-0.5wt%Cu target exhibits deformation textures after multidirectionally cold-rolled with a thickness reduction of 85% [59]. Post-annealing treatment at 250 °C for 120 min promoted the dominance of Cube-type texture with a concurrent reduction in the components of deformation texture. Compared to unidirectional rolling, the alloy targets subjected to large-strain multi-directional rolling developed an intensified <110> fiber texture. Notably, some residual deformation textures persisted after recrystallization annealing, indicating incomplete texture transformation [60]. For the multidirectionally rolling, texture evolution studies revealed temperature-dependent nucleation mechanisms. Annealing at low-temperature preferentially activated S-type ({123}<634>) and Cube-type nucleation. As the annealing time increases, other high activation energy orientation grains also grow. The weak initial texture intensity in the multidirectionally processed targets facilitated uniform grain growth across orientations, ultimately generating near-random recrystallization textures after full annealing.

The rolling reduction ratio is another critical factor influencing the crystallographic texture of sputtering targets. X.R. Li and co-workers [61] investigated ultra-high-purity Al-Cu alloy (99.999% Al and 0.3% Cu) processed through cold rolling with varying strain levels. At the strain of 60%, the deformed microstructure is dominated by Goss-type texture ({110}<001>). With the strain escalation to 80–98%, the typical rolling textures emerged where Brass component ({110}<112>) intensity surpassed S-type ({123}<634>) and Copper ({112}<111>) textures. Furthermore, with the increase in thickness reduction in cold rolling, the content of Brass texture increases. Post-annealing analysis revealed strain-dependent recrystallization behaviors: intermediate-strain specimens (60–80%) developed Cube ({001}<100>) and Cube_RD_ ({001}<310>) orientations, while high-strain alloys (>90%) subjected to low-temperature annealing (200–250 °C) preferentially formed Goss-oriented recrystallized grains. This strain–texture relationship establishes critical processing thresholds for orientation control in Al target manufacturing.

### 5.3. Dependence of Texture on the Sample Thickness

It is challenging to achieve a uniformly oriented aluminum alloy through thickness by conventional rolling, but this issue can be addressed through integrated thermomechanical processing strategies. Orientation analysis of the rolled Al-2at.%Nd alloys reveals significant texture gradients, with surface layers developing rotated cube textures {001}<110> while core regions maintain deformation textures ({123}<634>, {112}<111>, {011}<211>) [62]. Q. Li and co-workers [63] developed a hybrid processing route combining multi-directional forging with controlled hot rolling, successfully synchronizing surface-core orientation distributions in high-purity aluminum targets. This approach effectively improved the significant thickness inhomogeneity in high-purity aluminum targets prepared by cold rolling, addressing the issue of large orientation differences between the core and surface. It is helpful to prevent film thickness non-uniformity caused by differing sputtering rates between the surface and core during the sputtering process.

## 6. Summary

In summary, this review systematically consolidates advances in composition design, processing methodologies, and processing–microstructure relationships for aluminum-based targets. The addition of alloying elements, such as Si, Cu, Sc, and Nd, can effectively address key challenges in pure aluminum targets, including stress migration suppression and grain refinement. Thermomechanical processing, including forging, hot/cold rolling, annealing, and forging, emerges as a dominant strategy for microstructure control. Grain refinement could be achieved through mechanical rolling and recrystallization, with optimal deformation levels (70–90%) and annealing temperatures (250–350 °C) identified for balancing grain size uniformity and texture development. Crystallographic texture evolution exhibits a strong dependence on deformation modes (unidirectional vs. multidirectional rolling) and deformation degree, where Cube-type textures dominate after cold rolling and recrystallization annealing. A composite deformation strategy could successfully mitigate core-surface texture gradients and thus could be helpful in improving the homogeneity of the sputtered film. Apart from grain refinement, thermomechanical processing can also effectively refine alumina inclusions (such as Al_2_O_3_) that are easily formed in aluminum alloys, which remains a critical challenge in sputtering target manufacturing. Despite the great progress, critical knowledge gaps persist in understanding (dynamic) recrystallization mechanisms, texture evolution during multidirectional rolling, and scalability of hybrid processing routes. Additionally, the development of the ultrafine grain refinement technique with a grain size of less than <1 μm, realized by cryogenic undercooling treatment and severe plastic deformation methods (such as equal-channel angular extrusion), constitutes a critical research frontier in advanced target processing. The fabrication of high-performance aluminum sputtering targets requires the synergistic integration of solidification control, thermomechanical processing optimization, and strain-induced recrystallization regulation to achieve tailored microstructural characteristics. By establishing the relation between thermomechanical processing and microstructure, this review provides critical insights for designing high-performance aluminum targets tailored to next-generation thin-film applications.

## Data Availability

No new data were created or analyzed in this study. Data sharing is not applicable to this article.
